# Leucine-Rich Alpha-2-Glycoprotein as a non-invasive biomarker for pediatric acute appendicitis: a systematic review and meta-analysis

**DOI:** 10.1007/s00431-023-04978-2

**Published:** 2023-05-06

**Authors:** Javier Arredondo Montero, Blanca Paola Pérez Riveros, Oscar Emilio Bueso Asfura, María Rico Jiménez, Natalia López-Andrés, Nerea Martín-Calvo

**Affiliations:** 1 Department of Pediatric Surgery, Hospital Universitario de Navarra, 31008 Pamplona, Navarra, Spain; 2 Department of Preventive Medicine and Public Health, School of Medicine, University of Navarra, Pamplona, Spain; 3 Cardiovascular Translational Research. NavarraBiomed (Miguel Servet Foundation), Hospital Universitario de Navarra, Universidad Pública de Navarra (UPNA), IdiSNA, Pamplona, Spain; 4 IdiSNA, Instituto de Investigación Sanitaria de Navarra, Pamplona, Spain; 5 CIBER de Fisiopatología de la Obesidad y la Nutrición, Instituto de Salud Carlos III, Madrid, Spain

**Keywords:** Leucine-Rich Alpha-2-Glycoprotein, LRG1, Acute appendicitis, Pediatric, Children, Sensitivity, Specificity, Systematic review, Meta-analysis

## Abstract

The aim of this study was to analyze the diagnostic performance of Leucine-Rich Alpha-2-Glycoprotein (LRG1) in pediatric acute appendicitis (PAA). We conducted a systematic review of the literature in the main databases of medical bibliography. Two independent reviewers selected the articles and extracted relevant data. Methodological quality was assessed using the QUADAS2 index. A synthesis of the results, standardization of the metrics and 4 random-effect meta-analyses were performed. Eight studies with data from 712 participants (305 patients with confirmed diagnosis of PAA and 407 controls) were included in this review. The random-effect meta-analysis of serum LRG1 (PAA vs control) resulted in a significant mean difference (95% CI) of 46.76 μg/mL (29.26–64.26). The random-effect meta-analysis for unadjusted urinary LRG1 (PAA vs control) resulted in a significant mean difference (95% CI) of 0.61 μg/mL (0.30–0.93). The random-effect meta-analysis (PAA vs control) for urinary LRG1 adjusted for urinary creatinine resulted in a significant mean difference (95% CI) of 0.89 g/mol (0.11–1.66).

*Conlusion*: Urinary LRG1 emerges as a potential non-invasive biomarker for the diagnosis of PAA. On the other hand, due to the high between-study heterogeneity, the results on serum LRG1 should be interpreted with caution. The only study that analyzed salivary LRG1 showed promising results. Further prospective studies are needed to confirm these findings.

**Table:** 

**What is Known:** *• Pediatric acute appendicitis continues to be a pathology with a high rate of diagnostic error.* *• Invasive tests, although useful, are a source of stress for patients and their parents.*	
**What is New:** *• LRG1 emerges as a promising urinary and salivary biomarker for the noninvasive diagnosis of pediatric acute appendicitis.*

**Supplementary Information:**

The online version contains supplementary material available at 10.1007/s00431-023-04978-2.

## Introduction

The identification of novel tools that contribute to optimize the diagnosis of pediatric acute appendicitis (PAA) is a field of great scientific interest. Although pediatric ultrasound is available in most pediatric emergency departments, the diagnostic error rates of PAA reported in literature (both for negative appendectomy and for diagnostic delay in patients with PAA) are still very high and point to a need to for diagnostic optimization of this pathology [1–4].

The search for non-invasive diagnostic tests is of particular interest to all healthcare professionals working with children. In case of acute abdominal pain, blood tests can be an added source of stress for children and their parents. The possibility of using biological samples other than blood such as urine or saliva would represent a major advance in pediatrics. However, evidence on non-invasive diagnostic tests for PAA is scarce. A pilot study that assessed fecal calprotectin found inconsistent results [5]. Besides, the difficulty of obtaining a stool sample in the emergency department should also be considered. Urine 5-hydroxyindole acetic acid [6] and cortisol in hair [7] have also been evaluated. Considering that capillary sample processing requires specific equipment and trained personnel, the applicability in daily clinical practice of cortisol in hair is unfeasible.

Leucine-Rich Alpha-2-Glycoprotein (LRG1) is a 50 kDa glycoprotein that behaves as an acute phase reactant and has been strongly associated with systemic inflammatory processes, bacterial infections and neoplastic processes [8]. Predominantly synthesized in the liver, LRG1 is induced by different proinflammatory cytokines such as interleukin 1b, interleukin 6 and tumor necrosis factor alpha. Although its precise function is unknown, it is believed that following cell death that occurs in acute inflammatory or infectious processes, LRG1 contributes in the removal of cytochrome C that has been externalized to the bloodstream. Given its involvement in multiple autoimmune pathologies, it is also believed that LRG1 may have an immunomodulatory role [8] which would partially explain its participation in PAA. The aim of this paper was to synthesize the existing evidence on the performance of LRG1 obtained from different biological samples for the diagnosis of PAA.

## Methods

### Literature search and selection

We followed the Preferred Reporting Items for Systematic reviews and Meta-Analyses (PRISMA) guidance. We specifically designed and implemented a review protocol that was registered in the international prospective register of systematic reviews (PROSPERO ID CRD42023392220). Eligible studies were identified by searching in the main existing medical bibliography databases (PubMed, Medline, OVID, Web of Science, Scopus, Scielo and Cochrane library). Search terms used for medical subject headings and keywords were: (LRG OR ("LRG 1") OR (LRG-1) OR ("leucine-rich alpha-2-glycoprotein-1") OR ("leucine-rich alpha-2-glycoprotein") OR ("leucine-rich α-2-Glycoprotein 1")) AND ( paediatric OR pediatric OR children) AND (appendicitis OR ("acute appendicitis")) AND (serum OR plasma OR saliva OR salivary OR urine OR urinary). The search was last executed on 26.01.2023.

Inclusion and exclusion criteria are shown in Supplementary file 1. The selection of articles was made by JAM and BPR. Disagreements were resolved by consensus.

### Quality assessment

Methodological quality and risk of bias evaluation of the selected articles was performed with QUADAS2. Patient selection, index test, reference standard and flow and timing were evaluated in each selected article. Applicability concerns regarding patient selection, index test and reference standard were also assessed.

### Data extraction and synthesis

Two independent reviewers (JAM and BPR) extracted the relevant data from the selected articles following a standardized procedure. Extracted data included author, year of publication, study population (sample size, age range and sex distribution), PAA group and control group definitions, biological sample, mean and standard deviation (or median and interquartile range) for LRG1, statistical p-value for the between-group comparison, LRG1 area under the curve, cut-off value (if established), and its associated sensitivity and specificity. There were no disagreements between the reviewers after collating the extracted data. The metrics used in each study were reviewed and it was determined that standardization was necessary for the analysis. Conversion from ng/mL to μg/mL was performed when necessary.

### Meta-analysis

Medians (interquartile ranges) and medians (ranges) of LRG1 were transformed to means and standard deviations (sd) following a standard procedure [9]. Four random-effects meta-analyses were performed, one for serum LRG1 (control group vs PAA), one for unadjusted urinary LRG1 (control group vs PAA), one for urinary LRG1 adjusted for urinary creatinine (control group vs PAA) and the last one for urinary LRG1 adjusted for urinary creatinine (control group vs PAA) after excluding the work by Mahalik et al. The results were plotted in 4 forest plots. Between-study heterogeneity was assessed using the Chi^2^, Tau^2^ and I^2^ statistics.

## Results

**Fig. 1:**
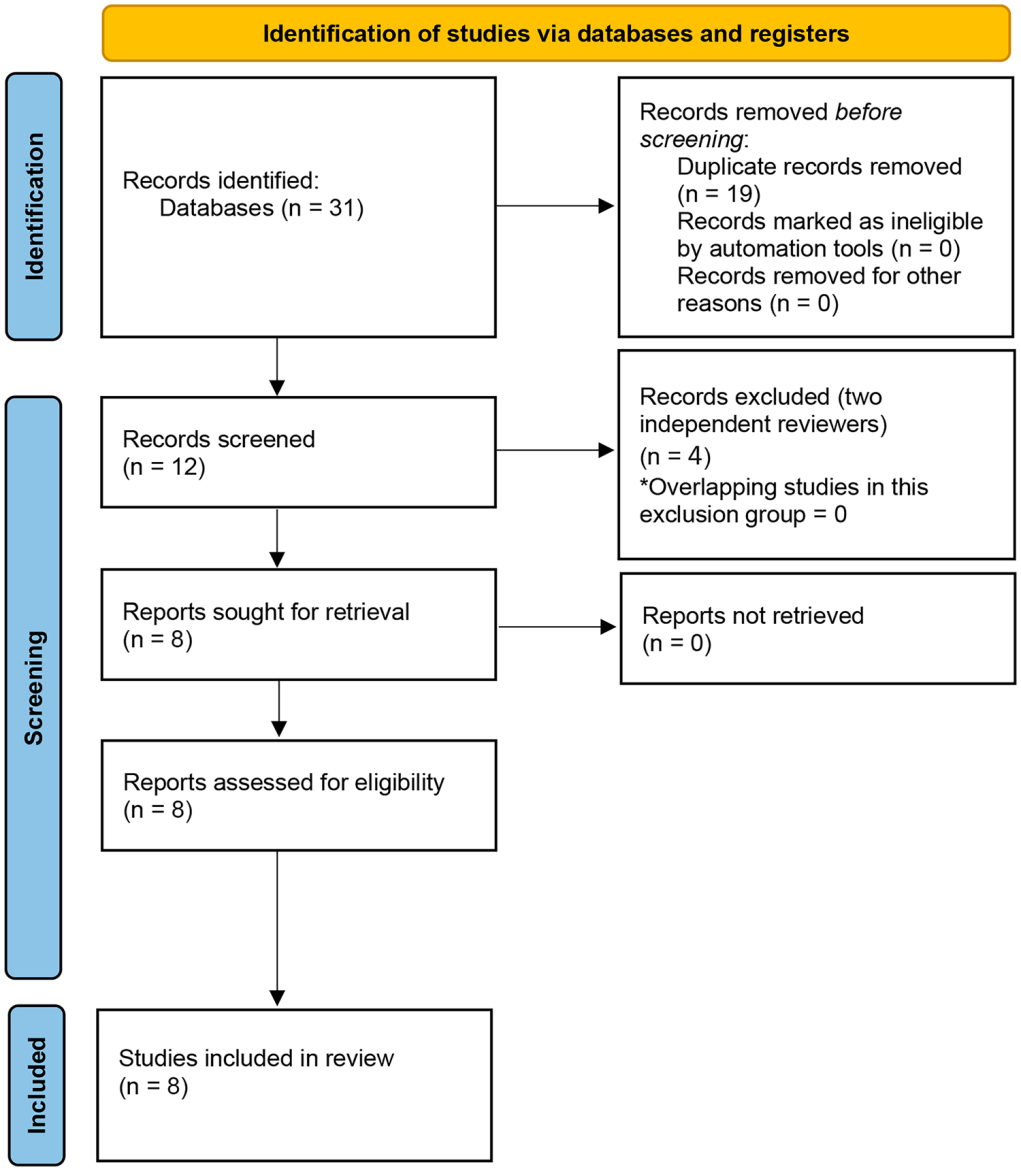
Flowchart of the search and selection process

The search returned 31 articles. Nineteen duplicates were removed. Among the remaining 12 articles, we excluded 4 following the inclusion and exclusion criteria (Fig. 1). This review finally included 8 studies with data from 712 participants (305 patients with confirmed diagnosis of PAA and 407 controls) [10–17].

**Fig. 2:**
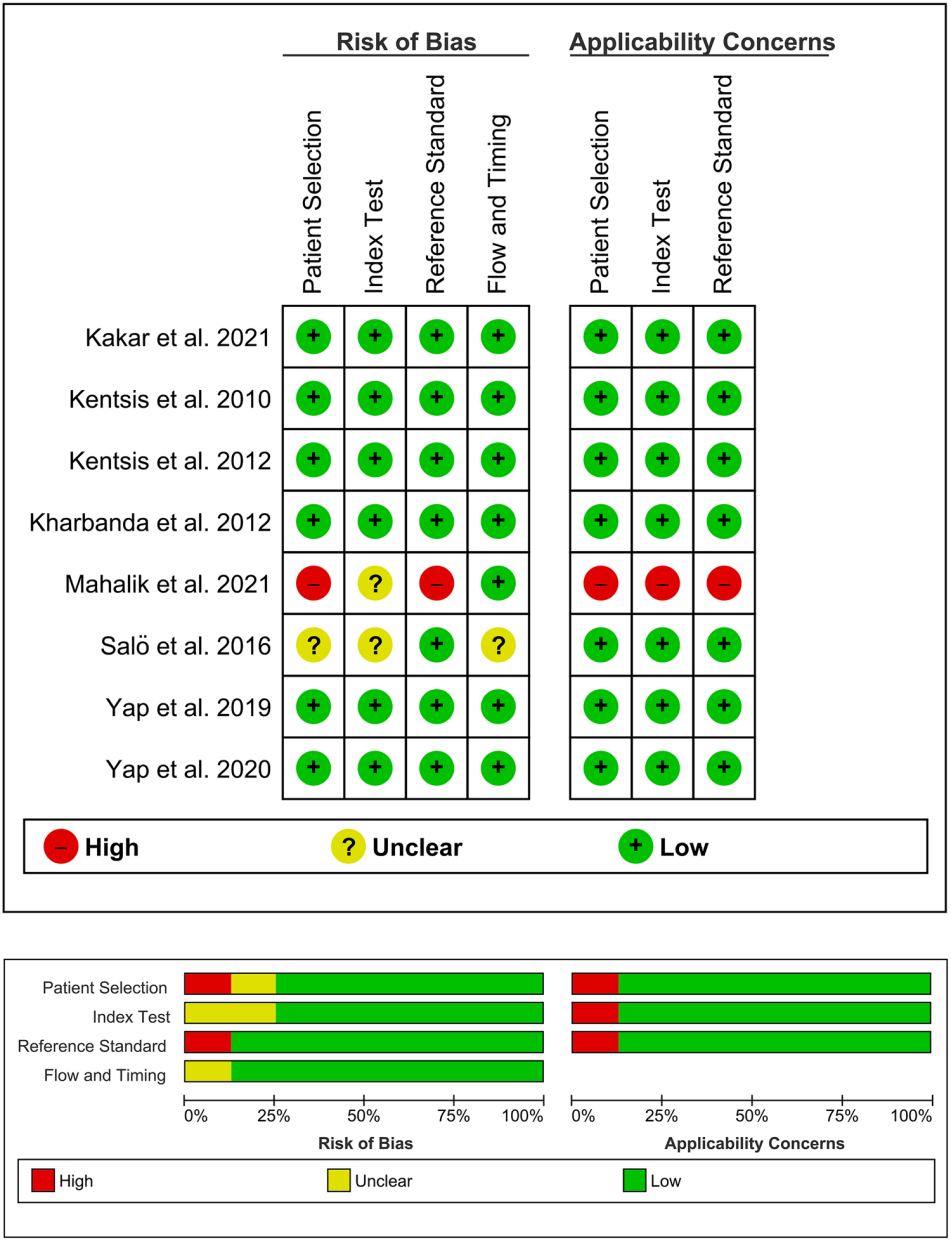
Graphical representation of the quality assessment of the diagnostic accuracy studies included in the review (QUADAS2)

The risk of bias in relation to the selection of patients was considered low in 6 of the 8 studies [10–12, 14, 15, 17], unclear in 1 of them [13] and high in the last one [16]. The risk of bias in relation to the index test was considered low in 6 of the studies [10–12, 14–17] and unclear in two of them [13, 16]. The risk of bias in relation to the reference standard was considered low in 7 of the studies [10–15, 17] and high in one of them [16]. The risk of bias in relation to flow and timing was considered low in 7 of the studies [10–12, 14–17] and unclear in one of them [13]. Regarding applicability concerns, the risk was estimated as low in all categories except for patient selection, for index test and for reference standard in 1 study [16]. The results of the QUADAS2 analysis are shown in Fig. 2.

### Serum Leucine-Rich Alpha-2-Glycoprotein

The data extracted from the studies that compared serum LRG1 levels is summarized in Table 1 [12, 17]. All studies were carried out between 2012 and 2021. One study was from Latvia [17] and the other was from the United States [12]. Both were prospective studies involving pediatric populations ranging from 3 to 18 years of age.

**Table 1:** Serum Leucine-Rich Alpha-2 Glycoprotein summary of publications included in this review

**Author**	**Study design**	**Age (Range)**	**Sex M/F**	**Total*N***	***N*** **in AA**	***N*** **in CG**	**Serum LRG1 AA** **(μg/mL)**	**Serum LRG1 CG (μg/mL)**	**P** **(CG vs AA)**	**P (NCAA vs CAA)**	**Cutoff μg/mL (CG vs AA)**	**AUC** **(AA vs CG)**	**AUC (NCAA vs CAA)**	**Sensitivity (%)**	**Specifity (%)**
Kharbanda et al. (2012) [12]	Prospective	3–18	92/84	176	58 (NCAA: 43 CAA: 15)	118	95396 (67198–144734)^a^*** [NCAA: 84763 (66728–135479)^a^*** CAA: 168546 (71497–202579)^a^***] **95.39 (67.20–144.73)**^a^**** **102.44 (58.94)**^b^	53593 (29898–117492)^a^*** **53.59 (29.90–117.49)**^a^**** **66.99 (65.74)**^b^	<0.001	0.05	40150*** 40.15****	0.69 (0.60–0.79)	-	100	35
Kakar et al. (2021) [17]	Prospective	7–17	89/64	153	97 (NCAA:45 CAA: 52)	56	NCAA: 88.12 (71.12–106.13)^a^ CAA: 70.56 (62.64–83.43)^a^ **NCAA: 88.46 (26.81)**^b^	34.08 (27.50–42.37)^a^ **34.65 (11.31)**^b^	< 0.001	< 0.001	51.69	0.95 (0.91–0.99)	0.69 (0.59–0.80)	93.8 (AA vs CG)	91.1 (AA vs CG)

Bold numbers: standarized metrics and estimated mean (sd) from median (IQR/range) as calculated by authors

*LRG1* Leucine-Rich Alpha-2 Glycoprotein, *AA* Acute appendicitis group, *CG* Control group, *NCAA* Non-complicated acute appendicitis, *CAA* Complicated acute appendicitis, *NS* non-statistically significant

***ng/mL; ****Conversion to μg/mL from ng/mL

^a^Median (Interquartile range)

^b^Mean (standard deviation) calculated from Median (Interquartile range)

All patients were recruited in the emergency department prior to diagnosis, and biological samples were obtained at the time of inclusion in the study.

The definition of “case” was inconsistent throughout the selected studies. Kharbanda et al. [12] confirmed the diagnosis of PAA using histopathology but the diagnosis of perforated PAA was based on the surgeon's findings, not on histopathologic criteria. Kakar et al. [17] reported having sent the appendectomy specimens for histological study but did not report any results. These authors classified patients into complicated and uncomplicated PAA based on microbiological culture from the peritoneal cavity. Significant variability was also identified in the definition of “control”, which was constituted either by patients with a formal suspicion of PAA (discarded after complementary tests/surgical evaluation) [12] or pediatric patients attended at the emergency department with no inflammatory process in the urinary, gastrointestinal or respiratory tract [17].

Regarding the method of determination, both authors used an ELISA kit following manufacturer's instructions. In the case of Kharbanda et al. [12] they did not specify the kit used, while Kakar et al. [17] reported having used a Novus Biologicals kit.

One study expressed serum LRG1 values in μg/mL [17] and the other one in ng/mL [12]. The results were presented as medians (interquartile range) [12, 17]. Both studies defined a specific cut-off point that ranged from 40.15 to 51.69 μg/mL and provided its associated sensitivity (from 93.8% to 100%) and specificity (from 35% to 91.1%) [12, 17]. The reported AUCs ranged from 0.69 to 0.95 [12, 17].

Both studies presented LRG1 values stratified by the histopathological appearance of the appendix (complicated vs. uncomplicated) [12, 17]. The differences in serum LRG1 values between complicated and uncomplicated PAA were statistically significant in both studies. One study analyzed the discriminatory capacity of LRG1 in that context and reported an area under the ROC curve (AUC) of 0.69 (95% CI 0.59–0.80) [17].

**Fig. 3:**
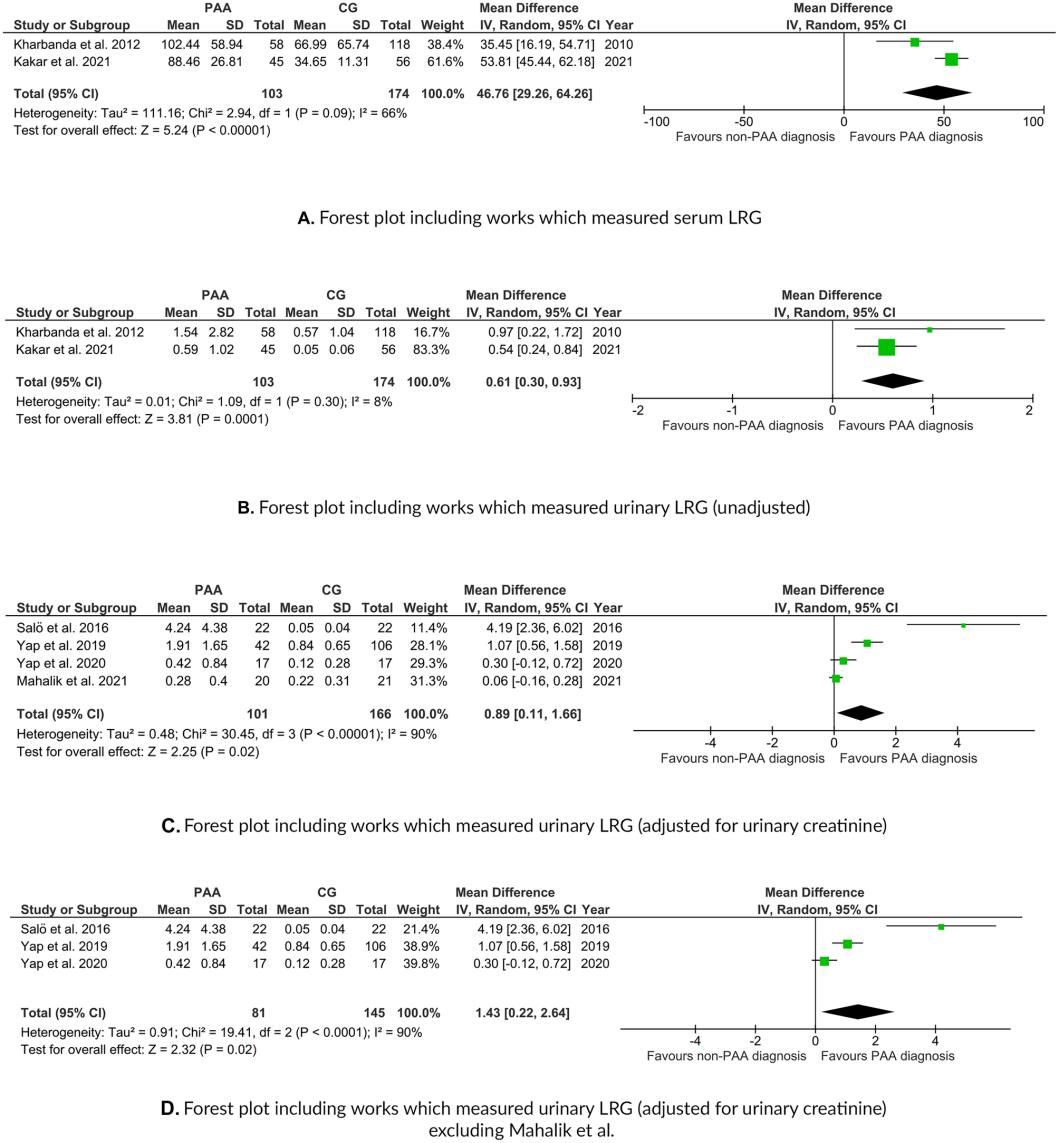
**A** Forest plot of the random-effects meta-analysis performed for serum LRG1 (PAA group vs. Control group). **B** Forest plot of the random-effects meta-analysis performed for unadjusted urinary LRG1 (PAA group vs Control group). **C** Forest plot of the random-effects meta-analysis performed for adjusted-for-creatinine urinary LRG1 (PAA group vs Control group). **D** Forest plot of the random-effects meta-analysis performed for adjusted-for-creatinine urinary LRG1 (PAA group vs Control group) excluding Mahalik et al

A standardization of metrics was performed before the quantitative analysis. The random-effect meta-analysis of those studies included 103 cases of PAA and 174 controls (Fig. 3). The pooled estimate resulted in a significant higher mean in the PAA group (difference [95% CI] of 46.76 μg/mL [29.26–64.26]) (p < 0.0001). The heterogeneity analysis showed a Chi^2^ value of 2.94 (p = 0.09) and an I^2^ value of 66%.

### Salivary Leucine-Rich Alpha-2-Glycoprotein

The data extracted from the single study that compared salivary LRG1 levels is summarized in Table 2 [15]. This was a prospective study carried out during 2020 in Singapore with a sample of 34 children aged between 4 and 16 years.

**Table 2:** Salivary Leucine-Rich Alpha-2 Glycoprotein summary of publications included in this review

**Author**	**Study design**	**Age (Range)**	**Sex M/F**	**Total*N***	***N*** **in AA**	***N*** **in CG**	**Salivary LRG1 AA** **(ng/μg)**	**Salivary LRG1 CG (ng/μg)**	**P** **(CG vs AA)**	**Cutoff ng/μg (CG vs AA)**	**AUC** **(AA vs control)**	**Sensitivity (%)**	**Specifity (%)**
Yap et al. (2020) [15]	Prospective	4–16	15/19	34	17	17	0.294 (0.161–0.457)^a^	0.126 (0.038,0.235)^a^	0.008	0.33	0.77 (0.60–0.93)	35.3	100

*LRG1* Leucine-Rich Alpha-2 Glycoprotein, *AA* Acute appendicitis group, *CG* Control group, *NCAA* Non-complicated acute appendicitis, *CAA* Complicated acute appendicitis, *NS* non-statistically significant

^a^Median (Interquartile range)

All patients were recruited in the emergency department prior to diagnosis and biological samples were obtained at the time of inclusion in the study.

The definition of “case” was the histopathological confirmation of appendicitis in the surgical specimen. The stratification of PAA in complicated and uncomplicated PAA was based on the presence of histological gangrene or histological appendiceal parietal perforation. The “control” group consisted of patients with formal suspicion of PAA (discarded after complementary tests/surgical evaluation) [15].

Regarding the method of determination, the authors reported having used an ELISA kit following manufacturer's instructions (IBL International, Takara, Japan).

Salivary LRG1 values were expressed in ng/μg and the results were presented as median (interquartile range). The authors reported significantly higher mean levels of salivary LRG1 in the PAA group than in the control group (p = 0.008) and an AUC of 0.77 (95% CI 0.60–0.93). The proposed cut-off point was 0.33 ng/μg and its associated sensitivity and specificity were 35.3% and 100% respectively.

### Urinary Leucine-Rich Alpha-2-Glycoprotein

The data extracted from the 8 studies that compared urinary LRG1 levels are summarized in Table 3 [10–17]. All studies were carried out between 2010 and 2021. One was from Latvia [17], 3 from the United States [10–12], 2 from Singapore [14, 15], 1 from Sweden [13] and 1 from India [16]. All studies were prospective and involved only pediatric populations aged between 3 and 18 years.

**Table 3:** Urinary Leucine-Rich Alpha-2 Glycoprotein summary of publications included in this review

**Author**	**Study design**	**Age (Range)**	**Sex M/F**	**Total *N***	***N* in AA**	***N* in CG**	**Urinary LRG1 AA** **(μg/mL)**	**Urinary LRG1 CG (μg/mL)**	**P** **(CG vs AA)**	**P (NCAA vs CAA)**	**Cutoff μg/mL (CG vs AA)**	**AUC** **(AA vs control)** **(95% CI)**	**Se (%)**	**Sp (%)**
Kentsis et al. (2010) [10]	Prospective	-	31/36	67	25 (CAA:4)	42	^−^	^−^	-		-	0.97 (0.93–1)**	-	-
Kentsis et al. (2012) [11]	Prospective	-	26/23	49	24 (NCAA: 18 CAA: 6)	25	3.9 (0.9-19.3)^b^ (interference adjusted)	0.3 (0.1-0-8)^b^ (interference adjusted)	-		-	0.80 (0.67–0.92)* 0.98 (0.96–1)**	-	-
Kharbanda et al. (2012) [12]	Prospective	3–18	92/84	176	58 (NCAA: 43 CAA: 15)	118	683.5 (122.3–3832.3)^b^***^(UA)^ [NCAA: 252.7 (106.7–2547.5)^b^***^(UA)^ CAA: 20576.8 (1750.4–38544.3)^b^***^(UA)^] **0.68 (0.12–3.83)**^b^****^(UA)^ **1.54 (2.82)**^d(UA)^	225.2 (46.5–1442.8)^b^***^(UA)^ **0.23 (0.05–1.44)**^b^****^(UA)^ **0.57 (1.04)**^e(UA)^	0.008^(UA)^	< 0.001^(UA)^	42***^(UA)^ 0.04****^(UA)^	0.63 (0.52–0.73)^(UA)^	100	23
Salö et al. (2016) [13]	Prospective	3–14	27/17	44	22 (NCAA: 14 CAA: 8)	22	0.078 (0.03–16.78)^X,c^ [NCAA: 0.06 (0.04–0.29)^X,c^ **4.24 (4.38)**^X,e^	0.014 (0.005–0.16)^X,c^ **0.05 (0.04)**^X,e^	< 0.001^X^	0.003^X^	0.036^X^ 0.26^(UA)^	0.86 (0.79–0.99)^X^ 0.65 (0.48–0.81)^(UA)^	86^X^ 64^(UA)^	73^X^ 50^(UA)^
Yap et al. (2019) [14]	Prospective	4–16	81/67	148	42 (CAA: 9 NA: 5)	106	0.22 (0.0003–7.20)^X,c^ **1.91 (1.65)**^X,e^	0.04 (0.0003–3.29)^X,c^ **0.84 (0.65)**^X,e^	0.014^X^	-	-	0.63 (0.53–0.72)^X^	-	-
Yap et al. (2020) [15]	Prospective	4–16	15/19	34	17 (NCAA:9 CAA: 8)	17	0.17 (0.026–1.068)^X,b^ **0.42 (0.84)**^X,d^	0.014 (0.002–0.353)^X,b^ **0.12 (0.28)**^X,d^	0.031^X^	-	1.5^X^	0.72 (0.54–0.90)^X^	17.7^X^	100^X^
Mahalik et al. (2021) [16]	Prospective	3–16	31/10	41	20 (NCAA: 2 CAA: 5 NOM: 13)	21	8.57 (8.02)^a(UA)^ 0.28 (0.40)^X^ MU not provided	6.68 (7.85)^a(UA)^ 0.22 (0.31)^X^ MU not provided	0.456 ^(UA)^ 0.59^X^	-	-	0.59 (0.41–0.77) not specified if adjusted or unadjusted	-	-
Kakar et al. (2021) [17]	Prospective	7–17	89/64	153	97 (NCAA:45 CAA: 52)	56	NCAA: 0.35 (0.05–1.38)^b(UA)^ CAA: 0.1 (0.03–0.73)^b(UA)^ **NCAA: 0.59 (1.02)**^**d**(UA)^ **CAA: 0.29 (0.53)**^**d**(UA)^	0.04 (0.02–0.10)^2(UA)^ **0.05 (0.06)**^**d**(UA)^	< 0.001^(UA)^	-	0.175^(UA)^	0.70 (0.62–0.79)^(UA)^	54.2	83.9

Bold numbers: standarized metrics and estimated mean (sd) from median (IQR/range) as calculated by authors

*LRG1* Leucine-Rich Alpha-2 Glycoprotein, *AA* Acute appendicitis group, *CG* Control group, *NCAA* Non-complicated acute appendicitis, *CAA* Complicated acute appendicitis, *NS* non-statistically significant, *Se* sensitivity, *Sp* specificity, *UA* Unadjusted to dehydration through urine creatinine, *NA* Negative appendectomies, *MU* Measurement units, *NOM* non-operative management

*ELISA; **High precision mass spectroscopy; ***ng/mL; ****Conversion to μg/mL from ng/mL X: adjusted to dehydration with urinary creatinine – LRG/Cr (g/mol) XX: adjusted to dehydration with urinary creatinine – units not provided

^a^Mean (standard deviation)

^b^Median (Interquartile range)

^c^Median (range)

^d^Mean (standard deviation) obtained from Median (IQR)

^e^Mean (standard deviation) obtained from median (Range)

In 7 of the 8 included studies [10–15, 17], patients were recruited in the emergency department prior to diagnosis, and biological samples were obtained at the time of inclusion in the study. One study [16] included patients from both the emergency department and an outpatient department.

The definition of “case” was consistent in six of the selected studies, given as the histopathological confirmation of PAA in the surgical specimen [10–15]. One study did not report the histopathologic diagnosis of PAA [16] and another study reported having sent the appendectomy specimens for histological study but did not report any results [17]. This was not the case for the definition of “control”, which was constituted either by pediatric patients attended at the emergency department with no suspected inflammatory process in the urinary, gastrointestinal or respiratory tract [17], patients with formal suspicion of PAA (discarded after complementary tests/surgical evaluation) [10–15] or patients with mesenteric lymphadenitis [16]. Regarding the stratification of PAA, 3 works classified the type of PAA based on histopathologic findings [10, 11, 15]. Another work stratified the type of PAA based on intraoperative findings reported by the surgeon (authors reported sending the sample for histological study, but they did not report the result) [13]. Another work used histopathology and intraoperative examination to determine the type of PAA [14]. Another work used clinical criteria exclusively [16]. Finally, the last work used microbiological culture findings for this classification [17].

Regarding the method of determination, 7 works reported having used a commercial ELISA kit following manufacturer's instructions [11–17] and 1 work, mass spectrometry/western blot [10]. Regarding the ELISA kits used, Kentis et al. and Yap et al. [11, 14, 15] used an IBL international kit, Kharbanda et al. [12], a Hycult Biotech kit (Uden, The Netherlands), Salö et al. [13], a Cusabio kit (Hubei province, China) and Mahalik et al. [16], an ASSAYPRO kit (USA).

One study expressed urinary LRG1 values as μg/mL [17] and another one as ng/mL [12]. Four studies presented urinary-creatinine-adjusted LRG1 values in g/mol [13–15]. The study by Mahalik et al. reported both crude and adjusted LRG1 values but they did not indicate the measurement units [16]. Our attempt to contact the authors to clarify this data was unsuccessful. Reviewing the ELISA kit used by the authors (ASSAYPRO ^©^) we saw that the determination was obtained in ng/mL. Crude urinary LRG1 values reported by Mahalik et al. differed greatly from those in previous works (4-fold higher values if microgram/milliliter was considered as the reported unit and 100-fold lower values if nanogram/milliliter was considered as the reported unit). Given the possibility that this data could be an error, we decided to exclude it from the unadjusted urinary LRG1 meta-analysis. We excluded from the meta-analysis 2 studies that either did not report urinary LRG1 values or only reported interference-adjusted urinary LRG1 values [10, 11]. The results were presented as means (standard deviations) [16], medians (interquartile ranges) [12, 15, 17] or medians (ranges) [13, 14]. Data presented by Salö et al. [13] was confirmed by the corresponding author via email.

Three studies defined a specific cut-off point for unadjusted urinary LRG1 between 0.04 and 0.26 μg/mL [12, 13, 17]. The associated sensitivity and specificity ranged from 54.2 to 100% and from 23.0 to 89.9% respectively. Two studies provided a cut-off point for urinary-creatinine-adjusted LRG1 between 0.036 and 1.5 g/mol [13, 15]. We cannot rule out the possibility that the latter is wrong since it is outside the range of values ​​presented by the authors. Our attempt to confirm this data did not obtain a response from the authors.

Two studies reported significant higher mean levels of unadjusted urinary LRG1 in the PAA group than in the control group [12, 17], whereas another study reported no significant differences [16]. Regarding urinary-creatinine-adjusted LRG1, 3 studies found significant higher mean levels in the PAA group compared to the control group [13–15], while one study reported no significant differences [16].

Three studies presented stratified values of urinary LRG1 by the histopathological appearance of the appendix (complicated vs. uncomplicated) [12, 13, 17]. The between-group comparison resulted in significant higher mean levels in the complicated group for both unadjusted [12] and adjusted LRG1 [13].

The random-effect meta-analysis for unadjusted urinary LRG1 included 103 cases of PAA and 174 controls [12, 17] (Fig. 3). The pooled estimate resulted in a significant higher mean in the PAA group (difference [95% CI] of 0.61 μg/mL [0.30–0.93]) (p = 0.0001). The heterogeneity analysis showed a Chi^2^ value of 1.09 (p = 0.30) and an I^2^ value of 8%. The random-effect meta-analysis of LRG1 adjusted for urinary creatinine LRG1 included 101 cases of PAA and 166 controls [13–16] (Fig. 3). The pooled estimate resulted in a significant mean difference [95% CI] of 0.89 g/mol [0.11–1.66] (p = 0.02). The heterogeneity analysis showed a Chi^2^ value of 30.45 (p < 0.001) and an I^2^ value of 90%. The fact that Yap et al. [15] reported statistically significant differences between groups but that in the meta-analysis their mean-difference crossed the null is due to our conversion of the reported median (interquartile range) to mean (standard deviation). The random-effect meta-analysis of LRG1 adjusted for urinary creatinine LRG1 (after excluding the work by Mahalik et al.) included 81 cases of PAA and 145 controls [13–15] (Fig. 3). The pooled estimate resulted in a significant mean difference [95% CI] of 1.43 g/mol [0.22–2.64] (p = 0.02). The heterogeneity analysis showed a Chi^2^ value of 19.41 (p < 0.001) and an I^2^ value of 90%.

## Discussion

This study systematically reviews the evidence on the role of LRG1 in the diagnosis of PAA. We synthetized the results of 8 prospective studies, including 305 patients with PAA and 407 controls, and performed 4 different meta-analyses that consistently showed significant higher mean values of serum, salivary and urinary LRG1 in the PAA group than in the control group. These findings are of great interest as they suggest that a non-invasive biomarker such as urinary LRG1 could be a useful tool for the diagnosis of PAA.

Regarding the studies that evaluated serum LRG1, the great difference in the AUCs for the discrimination between PAA and controls is striking: 0.69 (95% CI 0.60–0.79) [12]. vs. 0.95 (95% CI: 0.91–0.99) [17]. One possible explanation for this variability is the use of different definitions for the control group, which would also explain the high between-study heterogeneity observed in the meta-analysis (I^2^ = 66%). However, the AUCs for unadjusted urinary LRG1 in those same studies were similar: 0.63 (95% CI: 0.52–0.73) [12] and 0.70 (95% CI: 0.62–0.79) [17] and the heterogeneity observed in that meta-analysis was lower (I^2^ = 8%). One possible explanation is that Kakar et al. [17] overestimated the diagnostic performance of serum LRG1, and another possible explanation is that urinary LRG1 values show much less variability than serum values. However, considering the ranges reported by the other authors we believe that the latter is not true and that the analytical range of LRG1 is actually wide, both in patients with PAA and in controls.

Acute appendicitis constitutes a systemic metabolic-inflammatory insult which coupled with prolonged fasting, emesis, fever, and potential diarrhea may lead to the patient’s dehydration. A recent study found that patients with PAA presented higher values of capillary ketonemia than those with non-surgical abdominal pain [18]. To the best of our knowledge, no specific studies have evaluated the degree of dehydration in patients with PAA but Kharbanda et al. [12] postulated that urinary biomarkers of PAA should be adjusted for the degree of dehydration. In this study we found greater mean differences between groups but also wider confidence intervals in the meta-analysis of LRG1 adjusted for urinary creatinine than in the one of crude LRG1. In our opinion, further studies reporting both crude and adjusted urinary LRG1 values are needed to correctly interpret these differences and draw definitive conclusions.

Regarding creatinine adjusted urinary LRG1, 3 studies found significant differences between groups [13–15], whereas 1 study did not [16]. In our opinion, the latter [16] presents a high risk of bias because of the lack of histological confirmation of PAA in most of the cases and may have deviated our results to the null. Similarly, this work [16] did not report the units of measurement of urinary LRG1 nor did it state how they had calculated LRG1 adjusted for dehydration. The meta-analysis comprising the 4 studies showed very high between-study heterogeneity (I^2^ = 90%), and the meta-analysis after excluding the work of Mahalik et al. [16] maintained the same degree of statistical significance with a higher mean difference than previous analysis and similar values of heterogeneity (I^2^ = 90%). These results could be explained by several reasons. Firstly, LRG1 is adjusted for another biomarker (urinary creatinine), which means that two determinations are being performed on each patient, and the risk of measurement error is therefore doubled. Secondly, the included studies did not report isolated urinary creatinine values so we cannot rule out the presence of extreme values or that there is a high variability that conditions the adjustment. Last but not least, the investigators (Yap et al., Kakar et al., Mahalik et al.) reported having used different ELISA kits, which should be considered as an additional source of variability even though they followed the manufacturer's instructions and recommendations.

Given that LRG1 behaves as an acute phase reactant and it is related with other proinflammatory biomarkers [8, 19], its elevation in the context of PAA was not surprising. Besides, LRG1 is expressed by neutrophils, frequently involved in the early stages of PAA, and in the endothelial venules of the mesentery, including the mesoappendix [10, 17]. Interestingly, LRG1 is cleared in urine and its excretion rises in cases of renal failure and renal tubular injury [20, 21]. Although no studies have evaluated the role of LRG1 in prerenal (due to hypovolemia or dehydration) or renal failure, it should be considered that the diagnostic yield of urinary LRG1 in the context of PAA may be confounded in 1) patients with undiagnosed pre-existing renal pathology, and 2) patients with prerenal failure due to dehydration. Controlling for urinary creatinine may adjust the latter, but not the former. As it happens with other potential biomarkers of PAA, the main limitation of studies assessing the diagnostic performance of LRG1 is the lack of normal reference values, which severely hampers the use of this molecule in the routine clinical practice.

Obtaining a urine sample is conditioned by patients' urge to urinate which, in the case of acute appendicitis, is scarce due to the degree of dehydration that these patients usually present. In addition, the fact that these patients must remain fasting in case they undergo surgery further limits diuresis. Saliva, however, can be obtained at any time and in a simple way by using specific cotton wool pads. On the downside, saliva can be difficult to obtain in children under 2 years of age due to the size of the cotton swabs (standardized for adult population) and the risk of accidental ingestion. As we previously stated, the appeal of this study lies in the fact that its results suggest the possibility of using a non-invasive biomarker for the diagnosis of PAA. In this sense, the pilot study by Yap et al. [15] is of great relevance, since they found significant differences between groups for salivary LRG1. However, their findings are based on a pilot study and, while promising, need to be confirmed in studies with larger sample sizes before conclusions can be drawn.

An important aspect that is usually barely considered in diagnostic yield studies of acute appendicitis is the great variability for some parameters, such as leukocytes, during pediatric years [22]. Along with this, LRG1 has been associated with obesity in adolescence [23]. Therefore, future studies focused on the diagnostic performance of biomarkers in the context of PAA should consider adjustment for sociodemographic variables or present stratified results by age, sex and body mass index. Another critical aspect is the distinction between complicated and uncomplicated PAA, a field of enormous therapeutic, prognostic and socioeconomic relevance that is currently the subject of study by multiple working groups [24–26].

Despite our findings, we must acknowledge limitations. The scarcity of published literature, differences in study design and lack of consistency in the definition of control may have hampered our results. Lastly, we recognize that a significant mean difference between groups does not prove LRG1 has a good diagnostic yield in PAA. However, the included articles did not provide data to perform a diagnostic accuracy test meta-analysis. We encourage future research include information on true positives, true negatives, false positives and false negatives to allow the calculation of pooled sensitivity and specificity, as well as the representation of the AUC. On the other hand, we believe that this study has important strengths, such as the use of a rigorous and solid methodology based on the PRISMA and QUADAS2 guidelines [27, 28]. Furthermore, the age range and sex distribution of the samples described in this systematic review were reasonably homogeneous and representative of the pediatric population.

In conclusion, urinary LRG1 is a potential non-invasive biomarker for the diagnosis of PAA. Serum LRG1 could be a useful tool for the diagnosis of PAA, however due to the high heterogeneity between studies, the results should be interpreted with caution. Even though future prospective studies are needed to confirm these findings, the only study that analyzed salivary LRG1 showed promising results.

## Supplementary Information

Below is the link to the electronic supplementary material.

Supplementary file1 (DOCX 18 KB)

## Data Availability

The data used to carry out this systematic review are available upon request from the review authors.

## References

[1] Jones MW, Lopez RA, Deppen JG (2022) Appendicitis. 2022 Oct 24. In: StatPearls. Treasure Island (FL): StatPearls Publishing. (PMID: 29630245)

[2] Michelson KA, Reeves SD, Grubenhoff JA, Cruz AT, Chaudhari PP, Dart AH, Finkelstein JA, Bachur RG (2021) Clinical Features and Preventability of Delayed Diagnosis of Pediatric Appendicitis. JAMA Netw Open 4(8):e2122248. 10.1001/jamanetworkopen.2021.22248. (PMID: 34463745; PMCID: PMC8408667) 10.1001/jamanetworkopen.2021.22248 PMC8408667 34463745

[3] Goyal MK, Chamberlain JM, Webb M, Grundmeier RW, Johnson TJ, Lorch SA, Zorc JJ, Alessandrini E, Bajaj L, Cook L, Alpern ER, Pediatric Emergency Care Applied Research Network (PECARN) (2021) Racial and ethnic disparities in the delayed diagnosis of appendicitis among children. Acad Emerg Med 28(9):949–956. 10.1111/acem.14142. (Epub 2020 Oct 21. PMID: 32991770) 10.1111/acem.14142 32991770

[4] Michelson KA, McGarghan FLE, Patterson EE, Waltzman ML, Samuels-Kalow ME, Greco KF (2022) Clinician factors associated with delayed diagnosis of appendicitis. Diagnosis (Berl). 10.1515/dx-2022-0119. (Epub ahead of print. PMID: 36482753) 10.1515/dx-2022-0119 PMC10191871 36482753

[5] Sarsu SB, Erbagci AB, Ulusal H, Karakus SC, Bulbul ÖG (2017) The Place of Calprotectin, Lactoferrin, and High-Mobility Group Box 1 Protein on Diagnosis of Acute Appendicitis with Children. Indian J Surg 79(2):131–136. 10.1007/s12262-015-1441-2. (Epub 2016 Jan 14. PMID: 28442839; PMCID: PMC5386938) 10.1007/s12262-015-1441-2 PMC5386938 28442839

[6] Bosak Versic A, Glavan N, Bukvic N, Tomasic Z, Nikolic H (2016). Does elevated urinary 5-hydroxyindole acetic acid level predict acute appendicitis in children?. Emerg Med J.

[7] Gudjonsdottir J, Runnäs M, Hagander L, Theodorsson E, Salö M (2021). Associations of hair cortisol concentrations with paediatric appendicitis. Sci Rep.

[8] Zou Y, Xu Y, Chen X, Wu Y, Fu L, Lv Y (2022) Research Progress on Leucine-Rich Alpha-2 Glycoprotein 1: A Review. Front Pharmacol 12:809225. 10.3389/fphar.2021.809225. (PMID: 35095520; PMCID: PMC8797156) 10.3389/fphar.2021.809225 PMC8797156 35095520

[9] Wan X, Wang W, Liu J, Tong T (2014). Estimating the sample mean and standard deviation from the sample size, median, range and/or interquartile range. BMC Med Res Methodol.

[10] Kentsis A, Lin YY, Kurek K, Calicchio M, Wang YY, Monigatti F, Campagne F, Lee R, Horwitz B, Steen H, Bachur R (2010) Discovery and validation of urine markers of acute pediatric appendicitis using high-accuracy mass spectrometry. Ann Emerg Med 55(1):62–70.e4. 10.1016/j.annemergmed.2009.04.020. (Epub 2009 Jun 25. PMID: 19556024; PMCID: PMC4422167) 10.1016/j.annemergmed.2009.04.020 PMC4422167 19556024

[11] Kentsis A, Ahmed S, Kurek K, Brennan E, Bradwin G, Steen H, Bachur R (2012) Detection and diagnostic value of urine leucine-rich α-2-glycoprotein in children with suspected acute appendicitis. Ann Emerg Med 60(1):78–83.e1. 10.1016/j.annemergmed.2011.12.015. (Epub 2012 Feb 2. PMID: 22305331; PMCID: PMC3726720) 10.1016/j.annemergmed.2011.12.015 PMC3726720 22305331

[12] Kharbanda AB, Rai AJ, Cosme Y, Liu K, Dayan PS (2012) Novel serum and urine markers for pediatric appendicitis. Acad Emerg Med 19(1):56–62. 10.1111/j.1553-2712.2011.01251.x. (Epub 2012 Jan 5. PMID: 22221321; PMCID: PMC3261304) 10.1111/j.1553-2712.2011.01251.x PMC3261304 22221321

[13] Salö M, Roth B, Stenström P, Arnbjörnsson E, Ohlsson B (2016). Urinary biomarkers in pediatric appendicitis. Pediatr Surg Int.

[14] Yap TL, Fan JD, Chen Y, Ho MF, Choo CS, Allen J, Low Y, Jacobsen AS, Nah SA (2019). A novel noninvasive appendicitis score with a urine biomarker. J Pediatr Surg.

[15] Yap TL, Fan JD, Ho MF, Choo CSC, Ong LY, Chen Y (2020). Salivary biomarker for acute appendicitis in children: a pilot study. Pediatr Surg Int.

[16] Mahalik SK, Bandyopadhyay D, Tripathy BB (2021). Diagnostic accuracy of Leucine-rich α-2 glycoprotein (LRG) as a urinary biomarker in pediatric appendicitis: a prospective observational pilot study from Eastern India. Ann Pediatr Surg.

[17] Kakar M, Berezovska MM, Broks R, Asare L, Delorme M, Crouzen E, Zviedre A, Reinis A, Engelis A, Kroica J, Saxena A, Petersons A (2021). Serum and Urine Biomarker Leucine-Rich Alpha-2 Glycoprotein 1 Differentiates Pediatric Acute Complicated and Uncomplicated Appendicitis. Diagnostics (Basel).

[18] Arredondo Montero J, Bronte Anaut M, Bardají Pascual C, Antona G, López-Andrés N, Martín-Calvo N (2022). Alterations and diagnostic performance of capillary ketonemia in pediatric acute appendicitis: a pilot study. Pediatr Surg Int.

[19] Shirai R, Hirano F, Ohkura N, Ikeda K, Inoue S (2009)Up-regulation of the expression of leucine-rich alpha(2)-glycoprotein in hepatocytes by the mediators of acute-phase response. Biochem Biophys Res Commun 382(4):776–9. 10.1016/j.bbrc.2009.03.104. (Epub 2009 Mar 24. PMID: 19324010; PMCID: PMC7092932) 10.1016/j.bbrc.2009.03.104 PMC7092932 19324010

[20] Liu JJ, Pek SLT, Ang K, Tavintharan S, Lim SC, SMART2D study (2017) Plasma Leucine-Rich α-2-Glycoprotein 1 Predicts Rapid eGFR Decline and Albuminuria Progression in Type 2 Diabetes Mellitus. J Clin Endocrinol Metab 102(10):3683–3691. 10.1210/jc.2017-00930. (PMID: 28973352) 10.1210/jc.2017-00930 28973352

[21] Lee H, Fujimoto M, Ohkawara T, Honda H, Serada S, Terada Y, Naka T (2018). Leucine rich α-2 glycoprotein is a potential urinary biomarker for renal tubular injury. Biochem Biophys Res Commun.

[22] Li K, Peng YG, Yan RH, Song WQ, Peng XX, Ni X (2020). Age-dependent changes of total and differential white blood cell counts in children. Chin Med J (Engl).

[23] Alhammad R, Abu-Farha M, Hammad MM, Thanaraj TA, Channanath A, Alam-Eldin N, Al-Sabah R, Shaban L, Alduraywish A, Al-Mulla F, Rahman A, Abubaker J (2022). Increased LRG1 Levels in Overweight and Obese Adolescents and Its Association with Obesity Markers, Including Leptin, Chemerin, and High Sensitivity C-Reactive Protein. Int J Mol Sci.

[24] Arredondo Montero J, Antona G, Rivero Marcotegui A, Bardají Pascual C, Bronte Anaut M, Ros Briones R, Fernández-Celis A, López-Andrés N, Martín-Calvo N (2022) Discriminatory capacity of serum interleukin-6 between complicated and uncomplicated acute appendicitis in children: a prospective validation study. World J Pediatr 18(12):810–817. 10.1007/s12519-022-00598-2. (Epub 2022 Sep 16. PMID: 36114365; PMCID: PMC9617836) 10.1007/s12519-022-00598-2 PMC9617836 36114365

[25] Arredondo Montero J, Antona G, Bronte Anaut M, Bardají Pascual C, Ros Briones R, Fernández-Celis A, Rivero Marcotegui A, López-Andrés N, Martín-Calvo N (2022). Diagnostic performance of serum pentraxin-3 in pediatric acute appendicitis: a prospective diagnostic validation study. Pediatr Surg Int.

[26] Feng S, Wu P, Chen X (2014). Hyperfibrinogenemia in appendicitis: a new predictor of perforation in children. Pediatr Surg Int.

[27] Page MJ, McKenzie JE, Bossuyt PM, Boutron I, Hoffmann TC, Mulrow CD, Shamseer L, Tetzlaff JM, Akl EA, Brennan SE, Chou R, Glanville J, Grimshaw JM, Hróbjartsson A, Lalu MM, Li T, Loder EW, Mayo-Wilson E, McDonald S, McGuinness LA, Stewart LA, Thomas J, Tricco AC, Welch VA, Whiting P, Moher D (2021) The PRISMA 2020 statement: an updated guideline for reporting systematic reviews. BMJ 372:n71. 10.1136/bmj.n71. (PMID: 33782057; PMCID: PMC8005924) 10.1136/bmj.n71 PMC8005924 33782057

[28] Whiting PF, Rutjes AW, Westwood ME, Mallett S, Deeks JJ, Reitsma JB, Leeflang MM, Sterne JA, Bossuyt PM, QUADAS-2 Group (2011) QUADAS-2: a revised tool for the quality assessment of diagnostic accuracy studies. Ann Intern Med 155(8):529–36. 10.7326/0003-4819-155-8-201110180-00009. (PMID: 22007046) 10.7326/0003-4819-155-8-201110180-00009 22007046

