# Correction to: Leucine-Rich Alpha-2-Glycoprotein as a non-invasive biomarker for pediatric acute appendicitis: a systematic review and meta-analysis

**DOI:** 10.1007/s00431-023-05043-8

**Published:** 2023-06-02

**Authors:** Javier Arredondo Montero, Blanca Paola Pérez Riveros, Oscar Emilio Bueso Asfura, María Rico Jiménez, Natalia López-Andrés, Nerea Martín-Calvo

**Affiliations:** 1 Department of Pediatric Surgery, Hospital Universitario de Navarra, 31008 Pamplona, Navarra Spain; 2 Department of Preventive Medicine and Public Health, School of Medicine, University of Navarra, Pamplona, Spain; 3 Cardiovascular Translational Research. NavarraBiomed (Miguel Servet Foundation), Hospital Universitario de Navarra, Universidad Pública de Navarra (UPNA), IdiSNA, Pamplona, Spain; 4 IdiSNA, Instituto de Investigación Sanitaria de Navarra, Pamplona, Spain; 5 CIBER de Fisiopatología de la Obesidad y la Nutrición, Instituto de Salud Carlos III, Madrid, Spain

**Correction to: European Journal of Pediatrics** **https://doi.org/10.1007/s00431-023-04978-2**

In the original published version of the above article there were two typographical errors.

1. The text originally says "A standardization of metrics was performed before the quantitative analysis. The random-effect meta-analysis of those studies included **104** cases of PAA and 174 controls (Fig. 3)". However the correct number should have been **103** cases, not 104 (check Fig. 3)
2. On Table 3, on the Kakar et al. (2021) study, for Urinary LRG1 AA (μg/mL), the superindex (CAA) appears as a number and not as a letter. It should have been a "**d**“, not a "**4**“, since the legend of the table does not include numbers. The Table 3 has been corrected as follows.

**Table 3:** Urinary Leucine-Rich Alpha-2 Glycoprotein summary of publications included in this review

**Author**	**Study design**	**Age (Range)**	**Sex M/F**	**Total *N***	***N* in AA**	***N* in CG**	**Urinary LRG1 AA** **(μg/mL)**	**Urinary LRG1 CG (μg/mL)**	**P** **(CG vs AA)**	**P (NCAA vs CAA)**	**Cutoff μg/mL (CG vs AA)**	**AUC** **(AA vs control)** **(95% CI)**	**Se (%)**	**Sp (%)**
Kentsis et al. (2010) [10]	Prospective	-	31/36	67	25 (CAA:4)	42	^−^	^−^	-		-	0.97 (0.93–1)**	-	-
Kentsis et al. (2012) [11]	Prospective	-	26/23	49	24 (NCAA: 18 CAA: 6)	25	3.9 (0.9-19.3)^b^ (interference adjusted)	0.3 (0.1-0-8)^b^ (interference adjusted)	-		-	0.80 (0.67–0.92)* 0.98 (0.96–1)**	-	-
Kharbanda et al. (2012) [12]	Prospective	3–18	92/84	176	58 (NCAA: 43 CAA: 15)	118	683.5 (122.3–3832.3)^b^***^(UA)^ [NCAA: 252.7 (106.7–2547.5)^b^***^(UA)^ CAA: 20576.8 (1750.4–38544.3)^b^***^(UA)^] **0.68 (0.12–3.83)**^b^****^(UA)^ **1.54 (2.82)**^d(UA)^	225.2 (46.5–1442.8)^b^***^(UA)^ **0.23 (0.05–1.44)**^b^****^(UA)^ **0.57 (1.04)**^e(UA)^	0.008^(UA)^	< 0.001^(UA)^	42***^(UA)^ 0.04****^(UA)^	0.63 (0.52–0.73)^(UA)^	100	23
Salö et al. (2016) [13]	Prospective	3–14	27/17	44	22 (NCAA: 14 CAA: 8)	22	0.078 (0.03–16.78)^X,c^ [NCAA: 0.06 (0.04–0.29)^X,c^ **4.24 (4.38)**^X,e^	0.014 (0.005–0.16)^X,c^ **0.05 (0.04)**^X,e^	< 0.001^X^	0.003^X^	0.036^X^ 0.26^(UA)^	0.86 (0.79–0.99)^X^ 0.65 (0.48–0.81)^(UA)^	86^X^ 64^(UA)^	73^X^ 50^(UA)^
Yap et al. (2019) [14]	Prospective	4–16	81/67	148	42 (CAA: 9 NA: 5)	106	0.22 (0.0003–7.20)^X,c^ **1.91 (1.65)**^X,e^	0.04 (0.0003–3.29)^X,c^ **0.84 (0.65)**^X,e^	0.014^X^	-	-	0.63 (0.53–0.72)^X^	-	-
Yap et al. (2020) [15]	Prospective	4–16	15/19	34	17 (NCAA:9 CAA: 8)	17	0.17 (0.026–1.068)^X,b^ **0.42 (0.84)**^X,d^	0.014 (0.002–0.353)^X,b^ **0.12 (0.28)**^X,d^	0.031^X^	-	1.5^X^	0.72 (0.54–0.90)^X^	17.7^X^	100^X^
Mahalik et al. (2021) [16]	Prospective	3–16	31/10	41	20 (NCAA: 2 CAA: 5 NOM: 13)	21	8.57 (8.02)^a(UA)^ 0.28 (0.40)^X^ MU not provided	6.68 (7.85)^a(UA)^ 0.22 (0.31)^X^ MU not provided	0.456 ^(UA)^ 0.59^X^	-	-	0.59 (0.41–0.77) not specified if adjusted or unadjusted	-	-
Kakar et al. (2021) [17]	Prospective	7–17	89/64	153	97 (NCAA:45 CAA: 52)	56	NCAA: 0.35 (0.05–1.38)^b(UA)^ CAA: 0.1 (0.03–0.73)^b(UA)^ **NCAA: 0.59 (1.02)**^**d**(UA)^ **CAA: 0.29 (0.53)**^**d**(UA)^	0.04 (0.02–0.10)^2(UA)^ **0.05 (0.06)**^**d**(UA)^	< 0.001^(UA)^	-	0.175^(UA)^	0.70 (0.62–0.79)^(UA)^	54.2	83.9

Bold numbers: standarized metrics and estimated mean (sd) from median (IQR/range) as calculated by authors

*LRG1* Leucine-Rich Alpha-2 Glycoprotein, *AA* Acute appendicitis group, *CG* Control group, *NCAA* Non-complicated acute appendicitis, *CAA* Complicated acute appendicitis, *NS* non-statistically significant, *Se* sensitivity, *Sp* specificity, *UA* Unadjusted to dehydration through urine creatinine, *NA* Negative appendectomies, *MU* Measurement units, *NOM* non-operative management

*ELISA; **High precision mass spectroscopy; ***ng/mL; ****Conversion to μg/mL from ng/mL X: adjusted to dehydration with urinary creatinine – LRG/Cr (g/mol) XX: adjusted to dehydration with urinary creatinine – units not provided

^a^Mean (standard deviation)

^b^Median (Interquartile range)

^c^Median (range)

^d^Mean (standard deviation) obtained from Median (IQR)

^e^Mean (standard deviation) obtained from median (Range)

The original article has been corrected.

