# Supplementary material for: Leucine-Rich Alpha-2-Glycoprotein as a non-invasive biomarker for pediatric acute appendicitis: a systematic review and meta-analysis
Source: Eur J Pediatr. 2023 May 6;182(7):3033–44. doi: 10.1007/s00431-023-04978-2 (PMC10354117; doi:10.1007/s00431-023-04978-2)
Supplement: Supplementary file 1 — Supplementary file1 (DOCX 18 KB) [file 431_2023_4978_MOESM1_ESM.docx]

**Exclusion criteria**

-Case reports.

-Duplicate or overlapping studies.

-Reviews, systematic reviews, consensus guidelines.

-Languages other than English or Spanish.

-Studies with no surgical intervention.

-Studies with no population of interest.

-Patients older than 21 years old will be excluded. Nixed population studies in which there is no adequate stratification by subgroups of the pediatric population will be excluded.

-Studies conducted in immunocompromised patients.

-Studies conducted in patients with metastatic neoplastic disease and invasive abdominal neoplastic disease.

-Studies conducted in patients with acute or chronic kidney disease.

**Inclusion criteria**

-Prospective or retrospective observational original clinical studies evaluating the diagnostic accuracy of serum, salivary or urinary Leucine-Rich Alpha-2-Glycoprotein in relation to the reference standards for the diagnosis of appendicitis and/or for the discrimination between complicated and uncomplicated appendicitis in pediatric population.

-Diagnostic validation original studies evaluating the diagnostic accuracy of serum, salivary or urinary Leucine-Rich Alpha-2-Glycoprotein in relation to the reference standards for the diagnosis of appendicitis and/or for the discrimination between complicated and uncomplicated appendicitis in pediatric population.

**Supplementary file 1. Inclusion and exclusion criteria**
